# Myofibroblast differentiation and its functional properties are inhibited by nicotine and e-cigarette via mitochondrial OXPHOS complex III

**DOI:** 10.1038/srep43213

**Published:** 2017-03-03

**Authors:** Wei Lei, Chad Lerner, Isaac K. Sundar, Irfan Rahman

**Affiliations:** 1 Department of Environmental Medicine, University of Rochester Medical Center, Rochester, NY, USA; 2 Department of Respiratory Medicine, The First Affiliated Hospital of Soochow University, Suzhou, Jiangsu, 215006, China

## Abstract

Nicotine is the major stimulant in tobacco products including e-cigarettes. Fibroblast to myofibroblast differentiation is a key process during wound healing and is dysregulated in lung diseases. The role of nicotine and e-cigarette derived nicotine on cellular functions including profibrotic response and other functional aspects is not known. We hypothesized that nicotine and e-cigarettes affect myofibroblast differentiation, gel contraction, and wound healing via mitochondria stress through nicotinic receptor-dependent mechanisms. To test the hypothesis, we exposed human lung fibroblasts with various doses of nicotine and e-cigarette condensate and determined myofibroblast differentiation, mitochondrial oxidative phosphorylation (OXPHOS), wound healing, and gel contraction at different time points. We found that both nicotine and e-cigarette inhibit myofibroblast differentiation as shown by smooth muscle actin and collagen type I, alpha 1 abundance. Nicotine and e-cigarette inhibited OXPHOS complex III accompanied by increased MitoROS, and this effect was augmented by complex III inhibitor antimycin A. These mitochondrial associated effects by nicotine resulted in inhibition of myofibroblast differentiation. These effects were associated with inhibition of wound healing and gel contraction suggesting that nicotine is responsible for dysregulated repair during injurious responses. Thus, our data suggest that nicotine causes dysregulated repair by inhibition of myofibroblast differentiation via OXPHOS pathway.

Nicotine may be delivered systemically through various routes, such as oral or by transdermal diffusion. However, tobacco cigarettes and e-cigarettes (e-cig) are unique in their ability to deliver a nicotine laden aerosol to the lung^1^. Inhaled nicotine devices that are used for smoking cessation/replacement, such as e-cig causes throat irritation, nausea, dizziness and headache, but their long-term consequences on lung function is not known^2^,^3^ Nicotine binds with a family of nicotinic acetylcholine receptors (nAChRs), similar to acetylcholine (ACh)^4^,^5^. Fibroblasts and epithelial cells of the lung express abundant levels of nAChRs^4^,^5^,^6^,^7^. Moreover, these receptors trigger protease expression, mucin production, and smooth muscle contraction, which mediate airway obstruction in the pathogenesis of chronic obstructive pulmonary disease (COPD)^8^,^9^,^10^. Nicotine exerts immuno-modulatory responses that are both pro- and anti- inflammatory, but the responses are not well understood within an e-cig exposure setting. Whether or not nicotine can affect pulmonary inflammation and other cellular responses critical for tissue homeostasis via its receptor is not clear. Thus, it is possible that nicotine from e-cig or smoking cessation devices can lead to undesirable repercussions on cellular homeostasis and the pulmonary system.

Chronic airways disease and lung remodeling are well understood to occur as a consequence of smoking tobacco cigarettes, which in addition to nicotine deliver over 4000 individual compounds per puff. In contrast, the role of nicotine in specifically mediating biological effects that influence lung remodeling mechanisms is not known. The myofibroblast is a key effector cell for responding to lung injury and orchestrating repair, remodeling, and maintaining the extracellular matrix (ECM). Transforming growth factor beta 1 (TGF-β1) is a potent driver of myofibroblast differentiation from various progenitor cells, primarily resident fibroblasts^11^. In lung fibroblasts, deposition of collagen is highly dependent on TGF-β1 which does not enhance proliferation^12^. However, dysregulated TGF-β1 signaling is a prominent feature of pro-fibrotic or obstructive inflammatory airway pathologies in which abnormal collagen deposition and lung remodeling is typical^13^. Small airway remodeling plays a pivotal role in the development of COPD/emphysema^14^. These studies suggest that electronic nicotine delivery systems (ENDS) could cause adverse health effects in users, but there are no nicotine-derived e-cig studies conducted to date, focused on elucidating lung remodeling responses, in particular on lung fibroblasts. This implicates that e-cig delivering nicotine to the lung may affect myofibroblast differentiation in e-cig users, and hence might affect their ability to properly heal lung tissue by decreasing myofibroblast role in wound contraction. In this study, we examine the interplay between nAChRs activation by nicotine and TGF-β1 signaling in modulating the myofibroblast phenotypes and reveal a critical link between mitochondrial oxidative phosphorylation (OXPHOS) and TGF-β1 ability to drive myofibroblast differentiation.

## Methods and Materials

### Scientific Rigor and Reproducibility

We used a rigorous/robust and unbiased approach throughout the experimental plans (e.g. *in vitro* cells) and during analyzing the data so as to ensure that our data are reproducible along with by full and detailed reporting of both methods and analyzed data. All the key biological and/or chemical resources that are used in this study were validated and authenticated (methods and resources), and are of scientific standard from commercial sources. Our results adhere to NIH standards of reproducibility and scientific rigor.

### Cell culture and treatment

Human lung fibroblasts cells (HFL-1, ATCC) were cultured in Dulbecco’s modified Eagle’s medium and supplemented with 1% L-glutamine, 1% of penicillin/streptomycin, 1% of amino acid, and 10% fetal bovine serum (FBS)^15^. The cells (80% confluent) were washed gently with PBS, and media replaced without FBS. The cells were then treated with TGF-β1 (5 ng/mL), e-cigarette condensate (e-cig condensate, 0.1%), propylene glycol (PG)/vegetable glycerin (VG) condensate (2 μl/well), nicotine (100 μM or 1 mM, Sigma), mecamylamine (50 μM, Sigma), and a mitochondrial complex III inhibitor (antimycin A, 2 μM, Sigma) in a 6-well-plate for different time-points (0, 24, 48, 72 hrs). The supernatant from control and treated cells were used for slot blot analysis for Col 1A1 detection, and cell lysates used for Western blotting and other biochemical assays after protein quantification. Untreated cells/supernatants were used as controls. HFL-1 cells used in the experiments were in between 8 th and 12 th passages. The primary fetal lung HFL-1 cells were obtained from the commercial vendor (ATCC, VA), and cultured at 3.0% O_2_/5% CO_2_ at 37 °C in 100 mm dishes in growth media.

### Preparation of e-cig condensate

E-cigarette condensate (e-cig condensate) was freshly prepared by condensing vapors on dry ice generated from e-cig containing zero and 24.0 mg of nicotine with/without PG/VG from a local vendor. Vapors were generated using an eGO second generation e-cigarette device a refillable pen style ENDS (eGo Vision Spinner battery, China) and compatible clearomizer chamber (Anyvape, China) with 2.2 ohm heating element which was purchased from local retailers. The device was filled with nicotine with and without PG/VG to generate vapors/aerosols via a peristaltic pump connected to e-cig vaping device using a CSM-SSM machine (CH-Technologies Inc.). E-cig aerosols were drawn into the chamber every 30 seconds with a 4 second pulse^16^. The resulting condensed e-cig vapor was considered as 100%. The cells were treated with a final concentration of 0.25% e-cig condensate.

### Mitochondrial reactive oxygen species (MitoROS)/mitochondrial superoxide indicator (MitoSOX) assay by flow cytometry

Cells were treated with and without nicotine (100 μM) for 24 hrs, and mitochondria superoxide (as reactive oxygen specifies, ROS) assay was performed^17^ using MitoSOX by flow cytometry. After 24 hrs of treatment of cells with TGF-β1, antimycin A (complex III inhibitor), and TGF-β1 with antimycin A, treated cells were washed twice with PBS, and then added 800 μl MitoSOX-red (50 μg MitoSOX red in 13 μl DMSO) per well in 6-well plates which were then incubated at 37 °C for 10 minutes. Cell were washed 3 times with PBS, trypsinized, spun and washed again with PBS once. Finally, the cells were re-suspended in 150 μl PBS, and MitoSOX-MitoROS were measured by flow cytometry within 1 hr.

### α-SMA assay by immunofluorescence

HFL-1 cells were cultured in 6-well-plate for 24–72 hrs, and immunofluorescence was performed to assess HFL-1 differentiation. Cells were fixed with 2% paraformaldehyde for 10 minutes at room temperature, and then washed 3 times 5 minutes each with PBS, permeabilized with 0.5% Triton X-100 PBS for 15 minutes, further washed 3 times, 5 minutes each with PBS, and blocked with 0.3 M glycine, 0.1% Triton X-100, and 5% goat serum in PBS for 1 hr. The cells were incubated with α-SMA (Alexa Fluo-568) primary antibody dilution (1:1000) and fluorescein phalloidin dilution (1:500) in 1% BSA, 0.1% Triton X-100 in PBS overnight at 4 °C, washed 3 times 5 minutes each with PBS, and incubated with donkey anti-mouse secondary antibody dilution (1:1000) in 1% BSA, PBS for 1 hr. Cells were further washed 3 times 5 minutes each with PBS; and mounted with coverslips using 10 μl mounting reagent containing DAPI. Slides were dried for 5 minutes, and the images were taken by immunofluorescence microscope (Nikon).

### Western blotting

The protein concentrations of the whole cells lysates were measured using a BCA Protein Assay kit (Thermo Fisher). Equal amounts of protein from each sample were separated by sodium dodecyl sulfate–polyacrylamide gel electrophoresis (SDS-PAGE) and transferred to a nitrocellulose membrane. The membrane was incubated with anti-α-SMA antibody (1:1000 dilution) (Sigma), anti-OXPHOS antibody (Abcam, 1:2000 dilution), and anti-GAPDH antibody (Santa-Cruz, 1:1000 dilution) overnight at 4 °C. This primary antibody incubation was followed by incubation with HRP-conjugated anti-mouse (1:10,000 dilution) antibody or anti-rabbit (1:10,000 dilution) antibody as the secondary antibody for 1 hr at room temperature. The chemiluminescence was detected using the Bio-Rad ChemiDoc MP imaging system. Densitometric analyses of the band intensities were performed using ImageJ software (version 1.46; National Institutes of Health).

### Slot blot analysis

Six μl of cell culture supernatant/conditioned media was diluted in 30 μl PBS, then 25 μl mixture was applied to nitrocellulose membrane under gentle vacuum using a slot blot manifold (Harvard apparatus). The membranes were placed in blocking buffer (5% milk in blocking TPST buffer) for 1 hr at room temperature, incubated with primary antibody-soluble collagen type I alpha 1 (Col 1A1, Santa Cruz; 1:1000 dilution) overnight at 4 °C, washed 3 times 10 minutes each with wash buffer, and incubated with secondary antibody for 1 hr. The chemiluminescence on the membrane was detected using the Bio-Rad ChemiDoc MP imaging system. Densitometric analyses of the band intensities were performed using ImageJ software (version 1.46; National Institutes of Health).

### Wound healing scratch assay

The scratch assay was performed at wounding time intervals t = 0 hr, 24 hrs, 48 hrs, and 72 hrs for HFL-1 cells. Scratch/wound was inflicted using a pipette tip on a monolayer and cell migration was assessed by phase contrast microscopy. Quantification of wound closer as % (versus 100% control) relative distance between two scratch width of the edges (scratch wound) was performed and quantitated using ImageJ software.

### Gel contraction assay

HFL-1 cells were harvested and resuspended in culture media devoid of FBS. 400 μL resuspended media containing cells (8 × 10^4^ cells/well) was mixed with 160 μL cold collagen gel solution (3.75 mg/mL rat tail collagen I) and 8.6 μL 1 M NaOH. The prepared cell-collagen and NaOH mixture (500 μl) was added per well in a 24-well plate. After collagen polymerization, 600 μL of fresh media (without FBS) was added to each well containing gels. Cells were treated with TGF-β1 (5 ng/ml), nicotine (100 μM), TGF-β1 and nicotine, and 2, 3-butanedione monoxime (BDM, 10 mM), respectively. Gels were lifted from the well edges using sterilized probe immediately after the treatments. Gel images were taken by Bio-Rad ChemiDoc MP imaging system at the time point of 0, 24, 48, and 72 hrs and measured for contraction.

### Statistical Analysis

Statistical analyses were performed using SPSS 19.0 software (IBM, NY, USA). All of the data are reported as the mean ± SEM from at least three replicates. For all of the tests, p < 0.05 was considered statistically significant. To determine significance between two treatment groups, comparisons were made using an independent T-test, while ANOVA was used to analyze multiple groups, followed by a post-hoc Tukey HSD or LSD (if equal variance was assumed) and Dunnett T3 (if equal variance was not assumed) tests.

## Results

### Nicotine impairs the ability of lung fibroblasts to repair wound in scratch assay

**Figure 1:**
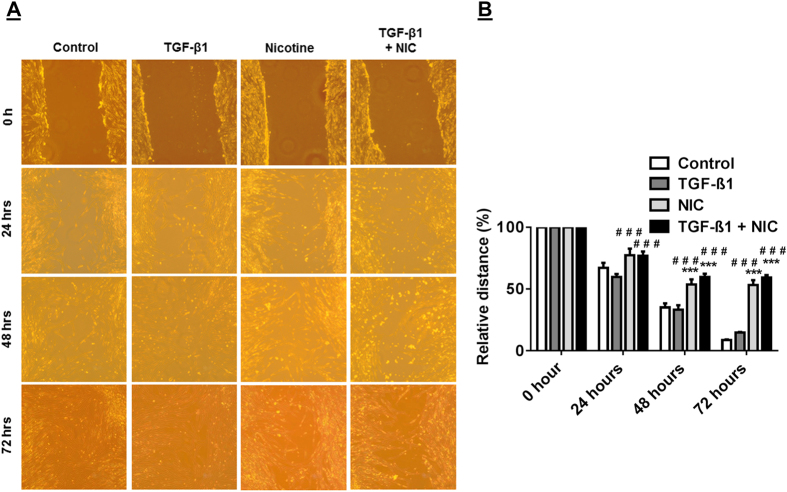
Nicotine impairs TGF-β1-mediated wound healing. Microscopic images of HFL-1 cultures following nicotine and TGF-β1 treatments (**A**) The scratch assay at initial wounding t = 0 h, 24 hrs, 48 hrs, and 72 hrs for HFL-1 cells incubated with nicotine, TGF-β1, nicotine and TGF-β1, or no treatment control. (**B**) Quantification of wound closer as % (versus 100% control) relative distance between two scratch width of the edges (scratch wound). Data are mean ± SEM. Data are a representative of at least three reproducible experiments. ***p ≤ 0.001, significant compared to control group; ^###^p ≤ 0.001, significant compared to TGF-β1 group. NIC, Nicotine.

**Figure 2:**
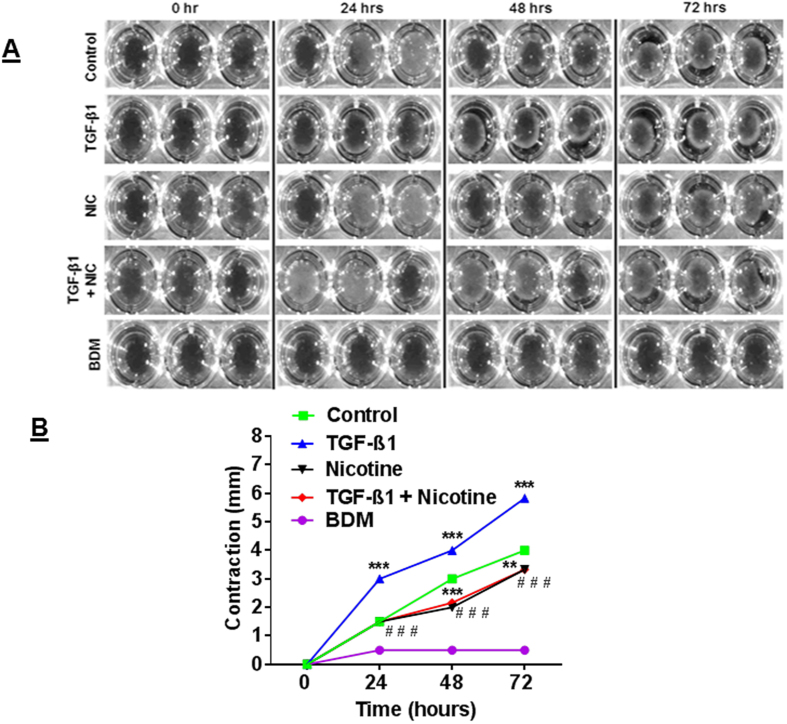
Nicotine impairs TGF-β1-mediated contractility. Photographs and quantitation of gel contraction from well edges following Nicotine and TGF-β1 treatments. HLF-1 cells were seeded into collagen gels and treated as indicated. (**A**) Representative images showing gel contraction. (**B**) Quantification of gel contractility by HFL-1 under indicated treatments. Contraction measured as mm gel substrate retreat from well edges. Data are mean ± SEM. Data are a representative of at least three reproducible experiments. **p ≤ 0.01, ***p ≤ 0.001, significant compared to control group; ^###^p ≤ 0.001, significant compared to TGF-β1 group. NIC, Nicotine; BDM, 2, 3-butanedione monoxime.

We sought to determine how nicotine might affect the ability of HFL-1 fibroblasts to activate a migratory phenotype in an *in vitro* scratch assay. The entire HFL-1 culture had reached confluence prior to introducing the scratch such that the severe reduction in cell cycle activity due to contact inhibition would slow proliferation from dominating and allow cells to migrate into the wound^18^. The progression of wound closing was monitored over a 72 hrs period in various conditions (Fig. 1A). A single nicotine treatment is most limiting in HFL-1 ability to close the wound by 72 hrs. TGF-β1 stimulation is similarly as effective compared to control conditions (see Materials and Methods) in healing. In contrast, nicotine severely limited wound closure despite TGF-β1 stimulation (Fig. 1B). Next, the ability of HFL-1 to undergo contractility, which can occur in part due to cells that have undergone myofibroblast differentiation in the wound healing process, was measured in various conditions. Single TGF-β1 stimulated cells consistently exhibited the highest contractile ability over other conditions throughout 72 hrs (Fig. 2A,B). By 48 hrs, nicotine reduced the contractility of the cells in addition to mitigating the ability for TGF-β1 to increase contractility. The myosin inhibitor BDM abolished contractility in HFL-1 (Fig. 2A). These results show nicotine interferes in lung fibroblast wound healing activity and suppresses TGF-β1 mediated enhancement of cell contractility.

### TGF-β1 induced fibroblast to myofibroblast differentiation is inhibited by nicotine

**Figure 3:**
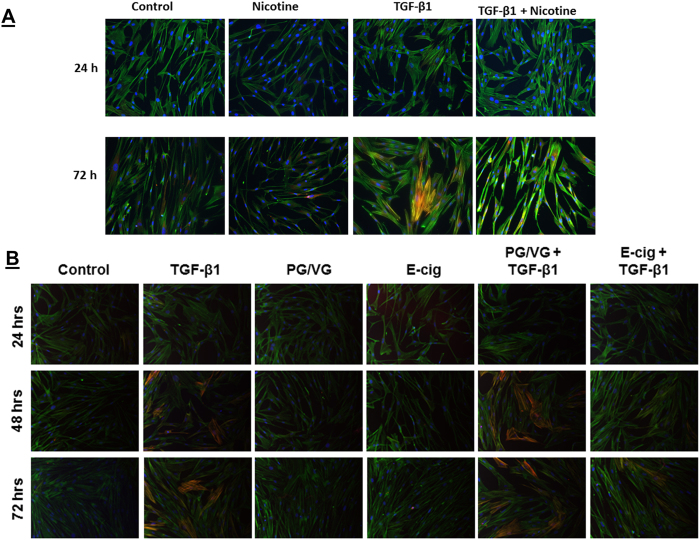
TGF-β1 induced fibroblast to myofibroblast differentiation is blocked by nicotine and e-cig condensate. Representative immunofluorescence microscopy images of HFL-1 for α-SMA (**A**) HFL-1 cells treated with TGF-β1, nicotine, TGF-β1 co-treated with nicotine, or no treatment control for 24 hrs or 72 hrs. The cell nuclei were stained with Dapi (blue) and actin with Phalloidin (green). Representative immunostaining of α-SMA-positive filamentous structures (yellow). (**B**) Treatment for 24 hrs, 48 hrs, or 72 hrs with TGF-β1, PG/VG, E-cig, TGF-β1 co-treated with PG/VG, or TGF-β1 with E-cig. Original magnification, 200x. Data are a representative of at least three reproducible experiments. E-cig, e-cigarette; PG, propylene glycol; VG, vegetable glycerin.

**Figure 4:**
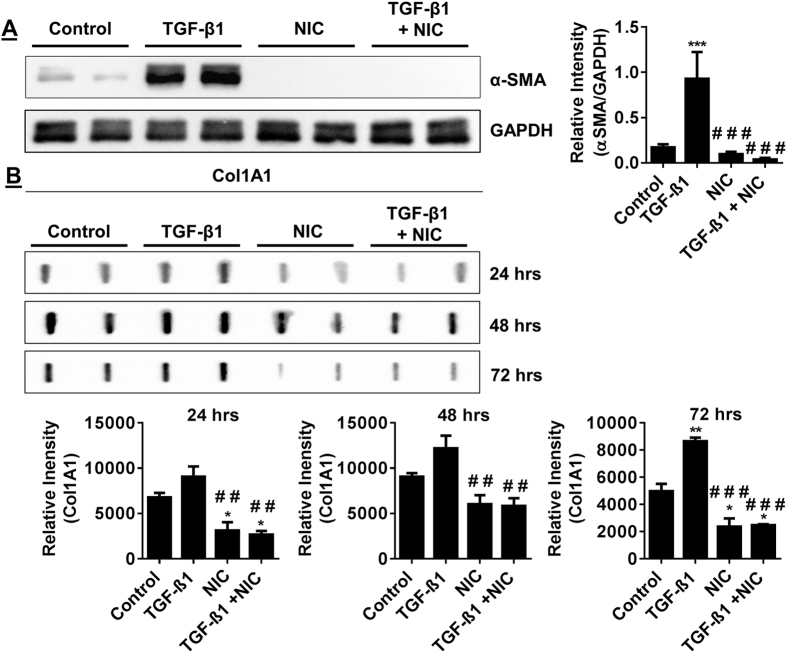
TGF-β1 induced α-SMA and Col 1A1 abundance in fibroblasts is suppressed by nicotine. Immunoblotting of α-SMA and Col 1A1 levels in HFL-1 incubated with TGF-β1, nicotine, TGF-β1 co-treated with nicotine (**A**) Representative immunoblot for α-SMA in HFL-1 lysates after cell treatments for 72 hrs; TGF-β1, nicotine and TGF-β1 with nicotine (**B**) Slot blot detection of Col 1A1 in conditioned media from HFL-1. Densitometry analysis represented in arbitrary units. Data are mean ± SEM. Data are a representative of at least three reproducible experiments. *p ≤ 0.05, **p ≤ 0.01, ***p ≤ 0.001 significant compared to control group; ^##^p ≤ 0.01, ^###^p ≤ 0.001 significant compared to TGF-β1 group. NIC, Nicotine.

To confirm that TGF-β1 induces fibroblast to myofibroblast differentiation, a critical step in the wound healing process, smooth muscle actin (α-SMA) and collagen (Col 1A1) secretion were measured in HFL-1 fibroblasts treated with TGF-β1. Induction of α-SMA expression was apparent by immunofluorescence after 72 hrs stimulation with TGF-β1 (Fig. 3A,B). In HFL-1 whole-cell lysates, α-SMA levels were increased in cells stimulated TGF-β1 for 72 hrs (Fig. 4A) with levels of Col 1A1 synthesis slightly elevated (Fig. 4B). TGF-β1 treatments for 24 hrs did not produce changes in α-SMA or Col 1A1 levels (Figs 3 and 4).

To determine how nicotine influences myofibroblast differentiation, HFL-1 cells were treated with nicotine alone or in combination with TGF-β1. Indication of α-SMA expression is not readily observable by immunofluorescence in HFL-1 after 24 hrs or 72 hrs treatment with nicotine (Fig. 3A). Whole cell lysates from untreated HFL-1 reveal a relatively low yet detectible amount of α-SMA compared to increased α-SMA levels in HFL-1 treated with TGF-β1 (Fig. 4A). In contrast, nicotine treatment effectively suppresses expression of basal α-SMA levels in HFL-1 lysates and further blocks induction of α-SMA when cells are co-stimulated both with nicotine and TGF-β1 (Fig. 4A). Nicotine also suppresses the ability of HFL-1 to secrete Col 1A1 with or without TGF-β1 at 72 hrs of treatment (Fig. 4B). These results show that nicotine can preclude HFL-1 from undergoing myofibroblast differentiation and represses TGF-β1 mediated myofibroblast differentiation by suppressing its ability to promote increased α-SMA expression and Col 1A1 abundance.

### E-cig condensate inhibits myofibroblast differentiation

**Figure 5:**
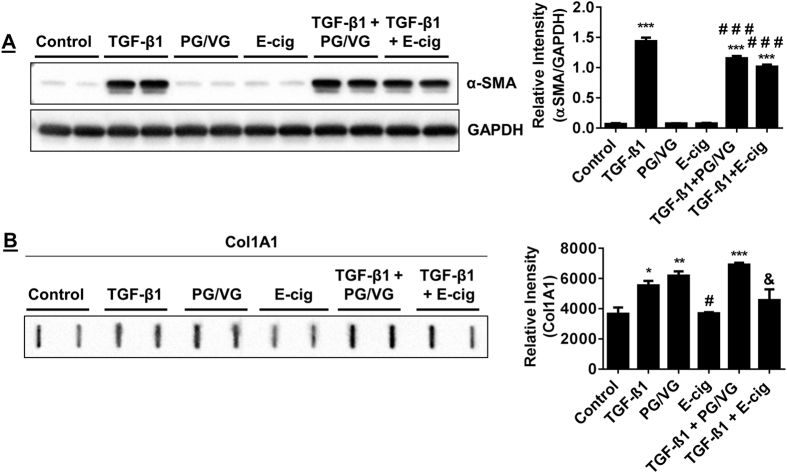
TGF-β1 induced α-SMA and Col 1A1 abundance in fibroblasts is suppressed by e-cig condensate. Immunoblotting of α-SMA and Col 1A1 levels in HFL-1 incubated with TGF-β1, PG/VG, E-cig, TGF-β1 with PG/VG, TGF-β1 co-treated with E-cig. (**A**) Representative immunoblot for α-SMA in HFL-1 lysates after cell treatments for 72 hrs; TGF-β1, PG/VG, E-cig, TGF-β1 co-treated with PG/VG, TGF-β1 co-treated with E-cig. (**B**) Slot blot detection of Col 1A1 in conditioned media from HFL-1. Densitometry analysis represented in arbitrary units. Data are mean ± SEM. Data are a representative of at least three reproducible experiments. *p ≤ 0.05, **p ≤ 0.01, ***p ≤ 0.001 significant compared to control group; ^#^p ≤ 0.05, ^###^p ≤ 0.001 significant compared to TGF-β1 group; & p ≤ 0.05 significant compared to TGF-β1 + PG/VG group. E-cig, e-cigarette; PG, propylene glycol; VG, vegetable glycerin.

Since nicotine can be delivered by inhalation of e-cig aerosols as effectively as tobacco smokers, we further explored how e-cig condensate affects myofibroblast differentiation when cells are co-stimulated with TGF-β1. HFL-1 stimulated with e-cig condensate, which includes manufacturer added nicotine, reduces the ability of TGF-β1 to exhibit robust α-SMA immunofluorescence staining after 72 hrs. Instead, these cells exhibit slight α-SMA positive staining compared to TGF-β1 stimulation alone (Fig. 3B). The stimulation of HFL-1 with e-cig condensate did not produce signs of α-SMA immunofluorescence (Fig. 3B). We also tested e-cig condensate that is devoid of added nicotine (PG/VG). In HFL-1 co-stimulated with PG/VG and TGF-β1, PG/VG did not affect TGF-β1 ability to produce α-SMA positive cells by immunofluorescence (Fig. 5A,B). Next, we measured the levels of α-SMA in HFL-1 whole cell lysates after cells were co-stimulated with e-cig condensate and TGF-β1. This treatment results in slightly reduced TGF-β1 mediated α-SMA expression compared to TGF-β1 alone, but increases α-SMA expression compared to unstimulated control (Fig. 5A). HFL-1 cells co-stimulated with TGF-β1 and PG/VG also results in a slight reduction in TGF-β1 mediated induction of α-SMA compared to TGF-β1 treatment alone with a TGF-β1 mediated increase in α-SMA compared to unstimulated cells (Fig. 5B). Neither e-cig nor PG/VG condensate treated HFL-1 resulted in altered levels of α-SMA compared to control group (Fig. 5A).

Col 1A1 release was increased in HFL-1 stimulated with PG/VG, TGF-β1, or, co-stimulated with TGF-β1 and PG/VG (Fig. 5B). In contrast, Col 1A1 release did not increase in HFL-1 stimulated with e-cig condensate (Fig. 5B). E-cig condensate prevented an increase in Col 1A1 when HFL-1 was co-stimulated with TGF-β1 and e-cig condensate (Fig. 5B).

These results show that e-cig condensate is capable of dampening TGF-β1 mediated induction of α-SMA, but is not effective at completely abolishing the ability of TGF-β1 to increase α-SMA. In addition, PG/VG independently induces Col 1A1 release suggesting it may promote myofibroblast differentiation when nicotine is absent. Lastly, e-cig condensate is effective at preventing TGF-β1 mediated Col 1A1 release.

### nAChRs mediates downstream effects of nicotine on myofibroblast differentiation

**Figure 6:**
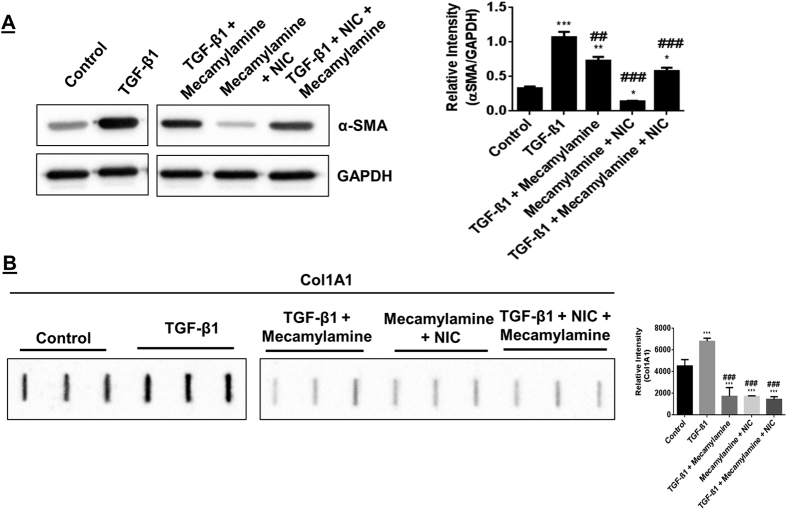
Inhibition of the nAChR protects against nicotine’s suppression of TGF-β1 mediated increase in α-SMA. Immunoblotting of α-SMA and Col 1A1 levels in HFL-1 following treatment with mecamylamine. (**A**) Representative immunoblot for α-SMA in HFL-1 lysates after cell treatments for 72 hrs; TGF-β1, TGF-β1 co-treated with mecamylamine, nicotine co-treated with mecamylamine, and TGF-β1 co-treated with nicotine and mecamylamine. (**B**) Slot blot detection of Col 1A1 in 72 hrs conditioned media from HFL-1. Gels/blots are cropped as displayed and gel demarcation is shown in boxes. Densitometry analysis represented in arbitrary units. Data are mean ± SEM. Data are a representative of at least three reproducible experiments. *p ≤ 0.05, **p ≤ 0.01, ***p ≤ 0.001 significant compared to control group; ^##^p ≤ 0.01, ^###^p ≤ 0.001 significant compared to TGF-β1 group. NIC, Nicotine.

Nicotine binding to nAChRs is an interaction that may influence cell physiology in a manner that leads to TGF-β1 mediated myofibroblast differentiation. To address this hypothesis, the nAChRs antagonist mecamylamine was used to treat HFL-1 cells stimulated with TGF-β1. Mecamylamine slightly reduced the ability of TGF-β1 to induce increased α-SMA. Next, nicotine stimulation was ineffective at significantly lowering α-SMA levels when mecamylamine is present. Finally, nicotine is unable to strongly suppress TGF-β1 mediated induction of increased α-SMA in the presence of mecamylamine although there is a measured reduction in α-SMA levels compared to single stimulation of cells by TGF-β1 (Fig. 6A). TGF-β1 is also impaired in its ability to promote Col 1A1 release in the presence of mecamylamine despite the absence of nicotine. Stimulation of cells with nicotine in addition to mecamylamine was sufficient to reduce Col 1A1 release compared to unstimulated control. Ultimately, the combination of mecamylamine and nicotine treatment also suppressed TGF-β1 mediated increase in α-SMA (Fig. 6B). These results suggest nicotine influences α-SMA expression through its binding to the nAChR. Inhibition of the nAChR also appears to be sufficient to impair Col 1A1 release with or without TGF-β1, but only slightly reduces TGF-β1 mediated induction of α-SMA.

### Mitochondrial complex III inhibition recapitulates nicotine suppression of myofibroblast differentiation

**Figure 7:**
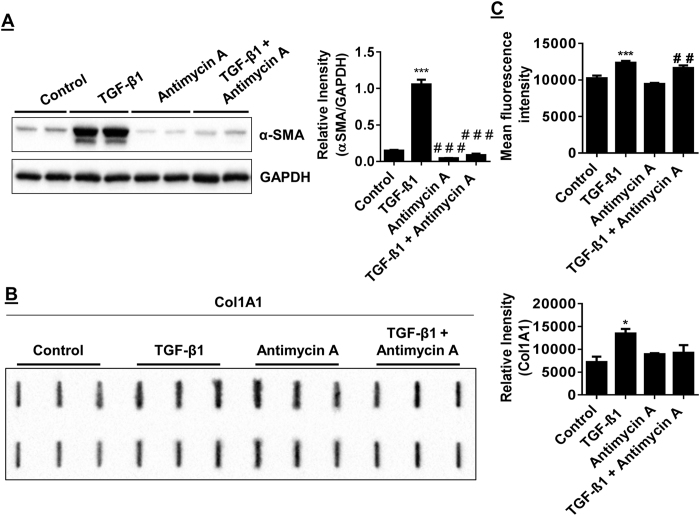
Inhibiting mitochondrial energetics recapitulates TGF-β1 alteration of α-SMA and Col 1A1. Immunoblotting of α-SMA and Col 1A1 levels in HFL-1 and FACS analysis detecting MitoSOX fluorescence. (**A**) Representative immunoblot for α-SMA in HFL-1 lysates after cell treatments for 72 hrs; TGF-β1, antimycin A, TGF-β1 with antimycin A. (**B**) Slot blot detection of Col 1A1 in 72 hrs conditioned media from HFL-1. (**C**) FACS analysis of MitoSOX staining in live HFL-1 cells treated with conditions as in (**A**). Densitometry analysis represented in arbitrary units. Data are mean ± SEM, and representative of at least three reproducible experiments. *p ≤ 0.05, ***p ≤ 0.001 significant compared to control group; ^##^p ≤ 0.01, ^###^p ≤ 0.001 significant compared to TGF-β1 group.

Nicotine has recently been reported to augment or disrupt properties of mitochondrial energetics^19^,^20^. We hypothesized TGF-β1 regulation is sensitive to changes in redox signaling since it can strongly potentiate increased ROS in lung fibroblasts. Thus, impairing mitochondrial OXPHOS at some level, would recapitulate what is observed when nicotine is used to suppress TGF-β1 mediated myofibroblast differentiation. To determine the role of OXPHOS activity in myofibroblast differentiation, HFL-1 cells were incubated for 24/72 hrs with either TGF-β1, complex III inhibitor antimycin A, or both. As expected, stimulation of HFL-1 with TGF-β1 increases α-SMA expression (Fig. 7A). Conversely, antimycin A treatment effectively suppresses expression of α-SMA levels (Fig. 7A). TGF-β1 mediated increase in Col 1A1 release was suppressed by co-treatment with antimycin A (Fig. 7B). Col 1A1 released remained unchanged when cells were treated with antimycin A alone (Fig. 7B).

**Figure 8:**
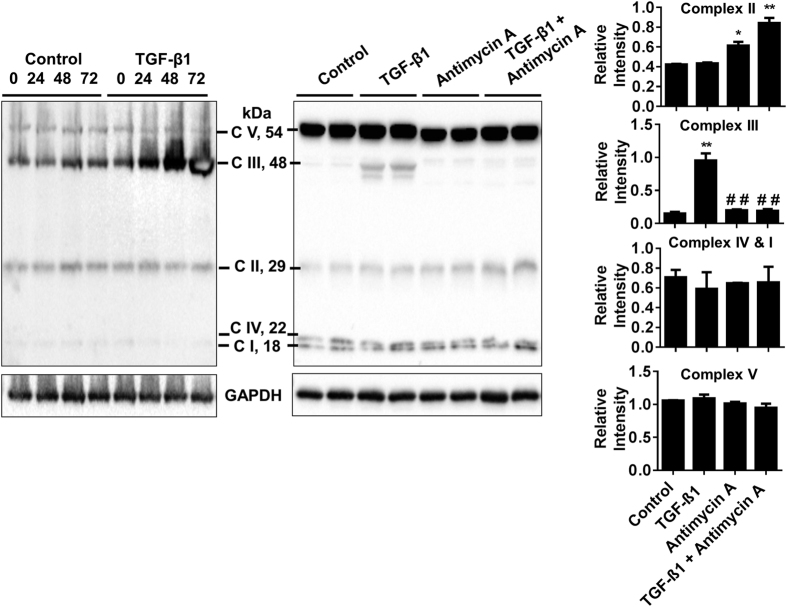
Inhibiting mitochondrial energetics recapitulates TGF-β1 mediated alteration of OXPHOS units. Representative immunoblot of mitochondrial complex subunits detected in HFL-1 lysate after incubation with TGF-β1 for indicated time points with TGF-β1 (left panel), with TGF-β1, antimycin A, TGF-β1 co-treated with antimycin A for 72 hrs (right panel). GAPDH is used as above in Fig. 7A for the same treatments. Densitometry analysis represented in arbitrary units. Data are mean ± SEM, and are a representative of at least three reproducible experiments. *p ≤ 0.05, **p ≤ 0.01 significant compared to control group; ^##^p ≤ 0.01 significant compared to TGF-β1 group.

**Figure 9:**
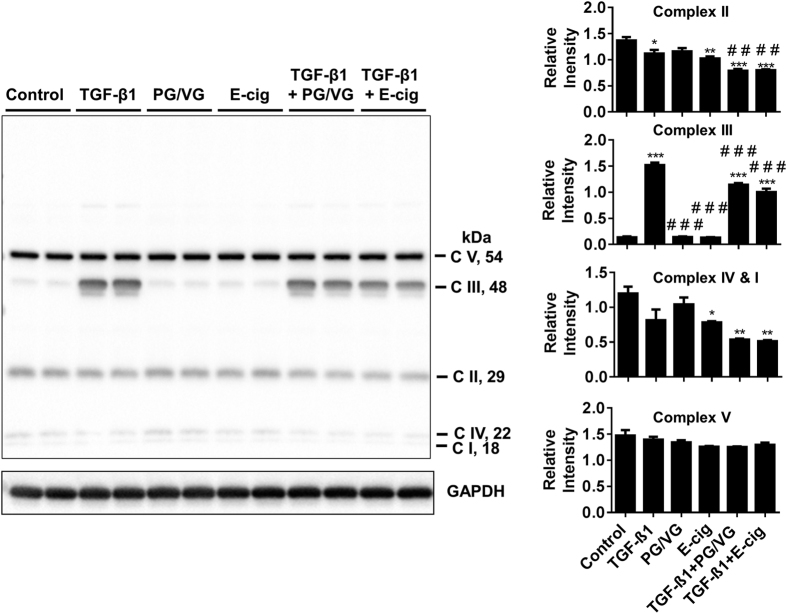
Inhibiting mitochondrial energetics recapitulates e-cig-induced alteration of OXPHOS units. Representative immunoblot of mitochondrial complex subunits detected in HFL-1 lysate after incubation with TGF-β1, E-cig, TGF-β1 co-treated with E-cig, PG/VG, E-cig, TGF-β1 co-treated with PG/VG, TGF-β1 co-treated with e-cig condensate for 72 hrs. Densitometry analysis represented in arbitrary units. Data are mean ± SEM, and are a representative of at least three reproducible experiments. *p ≤ 0.05, **p ≤ 0.01, ***p ≤ 0.001 significant compared to control group; ^##^p ≤ 0.01, ^###^p ≤ 0.001 significant compared to TGF-β1 group.

To determine how TGF-β1 and antimycin A influence MitoROS, HFL-1 was stained with MitoSOX mitochondrial superoxide indicator after incubation with TGF-β1, antimycin A, or both. TGF-β1 stimulation slightly increased levels of MitoROS. Antimycin A treatment alone did not result in a significant change in MitoROS, but was able to reduce TGF-β1 mediated increase in MitoROS when HFL-1 is incubated with co-treatments (Fig. 7C). The relative expression levels of mitochondrial complex III Core 2 subunit was increased by TGF-β1 stimulation, an effect which is abolished by co-treatment with both TGF-β1 and antimycin A. Single antimycin A treatment did not affect complex III Core 2 levels. Levels of Complex II subunit are increased by antimycin A and further increased by costimulation with TGF-β1. There were no changes in complex I, IV, or V subunit levels observed in any of the conditions (Fig. 8). Both e-cig and PG/VG condensate partially reduced TGF-β1 mediated increase in complex III Core 2 levels. Unlike antimycin A treatment, e-cig and PG/VG condensate reduced relative levels of the Complex II subunit when stimulated with TGF-β1. Single e-cig condensate is also sufficient to reduce Complex II levels (Fig. 9). Complexes I and IV exhibited reduced levels by e-cig condensate which was further reduced by stimulation with TGF-β1. No changes in complex V subunit levels were observed in any of the conditions (Fig. 9).

These results suggest that TGF-β1 mediated myofibroblast differentiation is sensitive to changes in mitochondrial energetics and shows that antimycin A was able to recapitulate suppression of myofibroblast differentiation by nicotine or e-cig condensate.

### nAChR influences TGF-β1 mediated changes in mitochondrial complex levels

**Figure 10:**
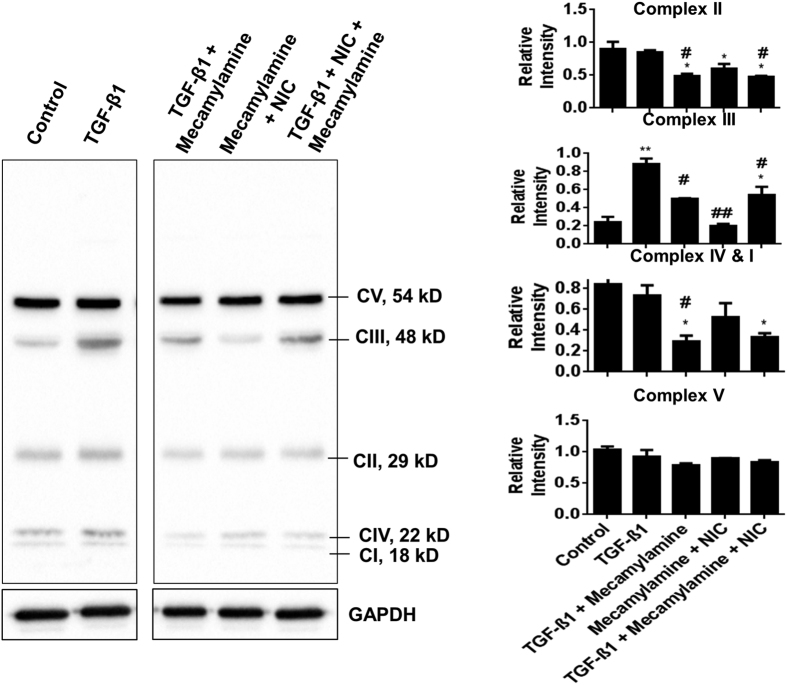
Inhibition of the nAChR protects against suppression of mitochondrial complex III subunit. Representative immunoblot of mitochondrial complex subunits in HFL-1 lysates after cell treatments for 72 hrs; TGF-β1, TGF-β1 co-treated with mecamylamine, nicotine co-treated with mecamylamine, TGF-β1 co-treated with nicotine and mecamylamine. Gels/blots are cropped as displayed and gel demarcation is shown in boxes. They are derived from the same samples/experiment and gels/blots were processed in parallel. GAPDH is used as above in Fig. 6 for the same treatments. Data are mean ± SEM, and are a representative of at least three reproducible experiments. *p ≤ 0.05, **p ≤ 0.01 significant compared to control group; ^#^p ≤ 0.05, ^##^p ≤ 0.01, significant compared to TGF-β1 group.

Nicotine binds with a family of nAChRs, similar to acetylcholine (ACh)^4^,^5^. These nAChRs are abundantly expressed in fibroblasts and epithelial cells of the lung^4^,^6^,^7^. Moreover, nAChR receptors trigger protease expression, mucin production, and smooth muscle contraction^8^,^9^, which mediate airway obstruction in the pathogenesis of COPD. Inhibiting the nAChR in HFL-1 with mecamylamine prevented TGF-β1 mediated increase in mitochondrial complex III Core 2 levels with or without nicotine. Treatment of cells with mecamylamine and nicotine together did not affect complex III Core 2 levels. However, they were reduced as compared to TGF-β1 treatment. There were slight changes in the levels of complex II, I, IV, or V subunits (Fig. 10). These results suggest nAChR signaling can interfere with TGF-β1 influence on expression of mitochondrial complex III Core 2 subunit.

## Discussion

Although the changes with fibroblast differentiation are well characterized, the mechanisms of the fibroblast differentiation particularly in response to nicotine derived from tobacco products are not studied. Further, little information is available in the exploration of mitochondrial bioenergetics associated with the fibroblast differentiation during stress response. The aim of this study was to determine the role of OXPHOS changes in their expression levels within fibroblasts treated with transforming growth factor-β1 (TGF-β1), a profibrotic cytokine known to activate fibroblasts into myofibroblasts.

Small airway remodeling plays a pivotal role in the development of COPD/emphysema^14^. In this study, we show nicotine affects fibroblast remodeling activity and this effect is recapitulated by perturbation of mitochondrial energetics. Inhibition of the nAChR partially protects against mitochondrial changes imparted by the presence of nicotine. We also studied the effect of nicotine and e-cigarette condensate on myofibroblast differentiation and other functional aspects of fibroblasts including mitochondrial OXPHOS, wound healing, and gel contraction at different time points. We show that both nicotine and e-cig condensate inhibited myofibroblast differentiation as shown by α-SMA staining as well as α-SMA and Col 1A1 abundance. Nicotine and e-cig condensate inhibited OXPHOS complex III accompanied by increased MitoROS, and this effect was augmented by complex III inhibitor antimycin A leading to inhibition of myofibroblast differentiation. These effects were associated with inhibited wound healing and gel contraction suggesting that nicotine is responsible for dysregulated repair during injurious responses.

We and others have recently shown that ENDS causes oxidative stress and inflammation in lung cells^16^,^17^,^21^, allergic response^22^, altered immunity^23^, impaired bacterial and viral clearance^24^. The data on myofibroblast differentiation and impaired wound healing as well gel contraction surmise that these effects may be due to oxidative stress and pro-inflammatory release in fibroblasts. Further, it is possible that e-cig condensate inhibits TGF-β1 release (unpublished observations), and hence have repercussions on the phenotype seen in this study.

The nAChRs are abundantly expressed in fibroblasts and epithelial cells of the lung^4^,^6^,^7^. Previous studies have identified nAChR subtypes located at the mitochondrial outer membrane^25^. Our data on nicotine mediated effects via its receptor antagonist suggest that the effect of nicotine is only partially mediated by its receptors at least in fibroblasts as the effect was not completely reversed using the nAChR antagonist mecamylamine. Nicotine present in e-cig condensate showed less potent effect on α-SMA compared to pure nicotine or antimycin A treatments, but still suppresses TGF-β1 mediated Col 1A1 release. From our results, it is interesting that mecamylamine (an antagonist of nAChRs) reduce Col 1A1 in the presence of TGF-β1 or nicotine, suggesting inhibition of nAChR is disrupting other potential regulators involved in promoting Col 1A1 through the nAChR receptor. There is a variety of subtype nAChR receptors that might differentially mediate downstream events that suppress TGF-β1 activity involved in promoting a myofibroblast phenotype, however the involvement of specific nAChR in particular alpha 3 and alpha 7 (predominantly in lung cells) requires further studies.

It has been shown that transforming growth factor-β1-induced epithelial to mesenchymal transition increases mitochondrial content in the A549 non-small cell lung cancer cell line. This was associated with ROS and profibrotic response^26^,^27^. TGF-β1-mediated differentiation of fibroblasts is associated with increased mitochondrial content and cellular respiration^28^. Further, TGF-β1 stimulates mitochondrial OXPHOS and generation of ROS in cultured mouse podocytes^29^. TGF-β1 induces senescence of bone marrow mesenchymal stem cells via increase of MitoROS production^30^. Thus, our data showing increased MitoROS during inhibition of myofibroblast differentiation involved mitochondrial stress response. Our data are consistent with these findings and show that nicotine and e-cig condensate affects myofibroblast differentiation. Suppressors of superoxide production from mitochondrial complex III have been described previously^31^. Complex III therefore, may have a critical role in orchestrating myofibroblast differentiation since mitochondrial reactive oxygen species regulate transforming growth factor-β signaling and during differentiation^32^,^33^.

Overall, we show that nicotine and antimycin A inhibit TGF-induced α-SMA and Col 1A1 in HFL-1. Nicotine disrupts OXPHOS in HFL-1, and antimycin A (a complex III OXPHOS inhibitor) augments the disruption, suggesting the involvement of mitochondrial energetics in myofibroblast differentiation. TGF-β1 induced mitochondrial complex III and nicotine/antimycin A inhibited OXPHOS complex III in fibroblasts. TGF-β1 induced MitoROS measured by MitoSOX, which was blocked by antimycin A. E-cig condensate disrupts OXPHOS in HFL-1, and antimycin A augments the disruption, further attesting the role of MitoROS and energetics in myofibroblast differentiation. Nicotine inhibits TGF-β1-induced α-SMA Col 1A1 in HFL-1 which is reversed by nicotinic receptor. Further nicotine impairs wound healing and gel contraction implicating the nicotine/e-cig effects on delayed wound healing and myofibroblast contractility. Thus, this study suggests that nicotine (an important component of e-cig) caused dysregulated repair via inhibition of myofibroblast differentiation via OXPHOS pathway. Recently, the TGF-β1 role in myofibroblast differentiation is shown to be accompanied by changes to cellular bioenergetics and requires that specific mitochondrial biogenesis factor Tfam is translocated into the mitochondria^33^. However, this requires further studies in response to nicotine and e-cigarettes. Though, our *in vitro* results reveal insights on myofibroblast differentiation imposed by nicotine, *in vivo* functional studies are required to confirm these findings using mouse models, such as exposing e-cig^16^ aerosols to mutants of specific nAChRs, which are in the interests of our future studies.

In conclusion, e-cig condensate inhibited myofibroblast differentiation. Nicotine inhibited α-SMA and Co11A1, and e-cig inhibited TGF-β1-induced α-SMA and Col 1A1 in HFL1 suggesting that the effect of e-cig and nicotine are similar, and hence implicating a pathophysiological role of these products in triggering profibrotic phenotypes not only in lung cells, but also in other cells/tissues of oral cavity leading to tissue (lung and sub-mucosal) fibrosis.

## Additional Information

**How to cite this article:** Lei, W. *et al*. Myofibroblast differentiation and its functional properties are inhibited by nicotine and e-cigarette via mitochondrial OXPHOS complex III. *Sci. Rep.* **7**, 43213; doi: 10.1038/srep43213 (2017).

**Publisher's note:** Springer Nature remains neutral with regard to jurisdictional claims in published maps and institutional affiliations.
